# Meloxicam, a Selective COX-2 Inhibitor, Mediates Hypoxia-Inducible Factor- (HIF-) 1*α* Signaling in Hepatocellular Carcinoma

**DOI:** 10.1155/2020/7079308

**Published:** 2020-03-24

**Authors:** Yinghong Zhou, Xiaofeng Dong, Peng Xiu, Xin Wang, Jianrong Yang, Lei Li, Zhongchao Li, Pengfei Sun, Xuetao Shi, Jingtao Zhong

**Affiliations:** ^1^School of Medicine and Life Sciences, University of Jinan-Shandong Academy of Medical Sciences, Jinan 250200, China; ^2^Department of Hepatobiliary Surgery, Shandong Cancer Hospital and Institute, Shandong First Medical University and Shandong Academy of Medical Sciences, Jinan 250117, China; ^3^Department of Hepatobiliary and Pancreatic Surgery Breast and Thyroid Surgery, The People's Hospital of Guangxi Zhuang Autonomous Region, Nanning 530021, China; ^4^Department of Hepatobiliary Surgery, Shandong Provincial Qianfoshan Hospital, The First Hospital Affiliated with Shandong First Medical University, Jinan 250014, China

## Abstract

Hepatocellular carcinoma (HCC) is regarded as a leading cause of cancer-related deaths, and its progression is associated with hypoxia and the induction of hypoxia-inducible factor (HIF). Meloxicam, a selective cyclooxygenase-2 (COX-2) inhibitor, induces cell death in various malignancies. However, the underlying mechanism remains to be elucidated in HCC, especially under hypoxic conditions. The alteration of COX-2 and HIF-1*α* oncogenicity was evaluated in HCC specimens by tissue microarray. Cell viability, angiogenesis assays, and xenografted nude mice were used to evaluate the effects of meloxicam, along with flow cytometry to detect the cell cycle, apoptosis, and mitochondrial membrane potential (*ΔΨm*) of HCC. qRT-PCR, Western blotting, immunofluorescence, immunohistochemistry, luciferase assay, and RNAi were carried out to determine the HIF-1*α* signaling affected by meloxicam. In this study, we showed that meloxicam exerts antiproliferative and antiangiogenesis efficacy *in vitro* and *in vivo* and causes disruption of mitochondrial membrane potential (*ΔΨm*), thus leading to caspase-dependent apoptosis under hypoxic environments. Exposure to meloxicam significantly reduced HIF-1*α* transcriptional activation and expression through sequestering it in the cytoplasm and accelerating degradation via increasing the von Hippel-Lindau tumor suppressor protein (pVHL) in HCC. These data demonstrated that inhibition of HIF-1*α* by meloxicam could suppress angiogenesis and enhance apoptosis of HCC cells. This discovery highlights that COX-2 specific inhibitors may be a promising therapy in the treatment of HCC.

## 1. Introduction

Hepatocellular carcinoma (HCC) ranks sixth among the most common carcinoma and is the fourth leading cause of cancer-related death worldwide [1]. Hepatitis virus infection (hepatitis B or C viruses), aflatoxins and aristolochic acid exposure, alcohol intake, and metabolic liver disease are considered the principal risk factors resulting in the development of HCC [2]. Surgical resection, orthotopic liver transplantation, and radiofrequency ablation are very effective therapeutic strategies in the early stage of HCC whereas most HCC patients are in an advanced stage of disease while diagnosed. Because the details of the molecular mechanisms of HCC progression are still unknown, there is currently a lack of effective systemic therapies, which causes the 5-year survival rate of advanced HCC to remain devastatingly low at 1% [3]. Consequently, there is an urgent need to better understand these mechanisms and develop new therapeutics for HCC treatment.

Hypoxia is an established characteristic of all solid tumors caused by aberrant vascularization and poor blood supply. In HCC, the degree of tumor hypoxia appears to be inversely related to the patient's prognosis. Also, it is usually resistant to traditional treatment [4, 5]. Hypoxia-inducible factor- (HIF-) 1, a heterodimeric transcriptional factor composed of HIF-1*α* and HIF-1*β*/ARNT subunits, is the best studied among HIF-*α* subunits and has a crucial role in mediating gene expression in order to maintain oxygen homeostasis. The HIF-1*α* subunit is rapidly degraded under normoxic conditions through two ways: von Hippel-Lindau tumor suppressor (pVHL) and factor inhibiting HIF1 (FIH1) [6, 7]. However, when the oxygen concentration declines, expression of HIF-1*α* exponentially increases which allows it to dimerize with the HIF-1*β* subunit to form hypoxia response elements (HREs) regulating targeted genes involved in the process of tumoral angiogenesis, proliferation, metastasis, and apoptosis of cancer cells [8, 9].

Cyclooxygenases (COXs) are an enzyme that are responsible for the formation of prostaglandin (PG) through rate limiting with three isoforms: COX-1 [10], COX-2 [11], and COX-3 (a splice variant of COX-1) [12]. It is widely accepted that COX-2 has an important role in the stimulation of inflammation and tumorigenesis in hypoxic cancer cells [13, 14], and the COX-2-selective inhibitor has been considered a potential candidate which can disturb the angiogenic signaling cascade upstream of HIF-1*α*-VEGF expression [15–17]. Our previous studies also indicated that meloxicam, a selective inhibitor of COX-2, has antiproliferative and proapoptotic effects in HCC [18–20]. However, the detailed mechanisms of meloxicam for treating HCC have not been fully explored, especially under a hypoxic microenvironment. The main purpose of this study was to explore the potential of meloxicam as therapy in HCC and to test the hypothesis that its effect on cell proliferation, angiogenesis, and related pathways might be involved in the treatment of meloxicam-induced apoptosis under hypoxic conditions.

## 2. Materials and Methods

### 2.1. Clinical Samples and Animal Experimental Protocols

Tissue microarrays (TMAs) from 90 HCC patients undergoing immunohistochemistry (HLivH180Su18) were obtained from Outdo Biotech Co., Ltd. (Shanghai, China). Ethical evidence provided by the company confirmed the consent procedures approved by the local ethics committee. The ethical statement and experiment protocol were approved by the institutional research ethics committee of Shandong Cancer Hospital and Institute. Expressions of COX-2 and HIF-1*α* in TMAs were detected by immunohistochemistry (IHC). Survival time was calculated from the date of surgery to the end of the follow-up or the date of death.

All animal experimental protocols (SDTHEC-201912007) were carefully checked and approved by the institutional research ethics committee of Shandong Cancer Hospital and Institute, and the methods were described in our previous studies [5, 21]. Huh-7 and Hep3B cells (5 × 10^6^/0.1 ml) in PBS were inoculated into the dorsal area near the front leg of 4-week-old BALB/c nude mice (10 mice per cell type) (HFK Bioscience Company, Beijing, China). The observation of mice continued until tumors developed to a size of 100 mm^3^. Then, the mice were randomly divided into four groups (5 in each group) Two groups were mice inoculated with Huh-7 cells, and two groups were mice inoculated with Hep3B cells. The experimental groups were managed by intraperitoneal injection of meloxicam (30 mg/kg) diluted in PBS every two days, while the control group was managed by an identical volume of 0.9% normal saline (0.9% NS). The tumors were harvested at the end of the experiments.

### 2.2. Reagents and Cell Culture

Meloxicam was acquired from Merck Millipore (Darmstadt, Germany). Cycloheximide, Z-VAD-FMK, JC-1, and MG-132 were purchased from Sigma-Aldrich (San Diego, USA). Primary antibodies to HIF-1*α*, VEGFA, von Hippel-Lindau, PARP1, cleaved PARP1, Ki-67, CD31, and GAPDH were obtained from Abcam (Cambridge, UK). Primary antibodies to caspase-3, cleaved caspase-3, caspase-9, cleaved caspase-9, and histone H3 were obtained from Cell Signaling Technologies (Danvers, MA).

The 2 human HCC cell lines, Huh-7 and Hep3B, and the normal human liver cell line, L-02, were obtained from the American Type Culture Collection (ATCC, Manassas, VA) and preserved in our laboratory. The cells were routinely cultured in RPMI 1640 medium (Gibco, Grand Island, NY, USA) supplemented with 10% fetal bovine serum (Gibco) and 1% antibiotics at 37°C in 95% air and 5% CO_2_. Human umbilical vein endothelial cells (HUVECs) were purchased from ScienCell Research Laboratories (Carlsbad, CA, USA). The cells were cultured in endothelial cell medium (ECM, ScienCell Research Laboratories) at 37°C in 95% air and 5% CO2. For the hypoxia experiments, Huh-7 and Hep3B cells were cultured in a hypoxic chamber (Billups-Rothenberg, Inc.) with 1% O_2_, 5% CO_2_, and 94% nitrogen.

### 2.3. Flow Cytometric Analysis of Cell Cycle, Apoptosis, and Mitochondrial Membrane Potential (*ΔΨ*m)

Cells (4 × 10^5^/well) were seeded with culture medium in a 6-well plate, incubated at 37°C for 24 h, and then incubated with fresh medium with various concentrations of meloxicam (0 to 80 *μ*M) combined with or without Z-VAD-FMK (50 *μ*M) in a hypoxic chamber. After washing twice with cold PBS and resuspending with binding buffer, the ANXA5-FITC/PI Detection Kit (BD Biosciences, San Jose, CA) was used to analyze apoptotic cells and the cell cycle distribution by flow cytometry according to the manufacturer's instruction. For JC-1 staining, cells were resuspended in PBS, containing 0.1 *μ*M JC-1, and were incubated at 37°C for 15 min in the dark. Then, cells were detected with flow cytometry.

### 2.4. Quantitative Real-Time RT-PCR (qRT-PCR) Analysis

The detailed methodology was described in our previous study [22]. Briefly, total RNA was prepared with Trizol (Invitrogen, Carlsbad, USA), and cDNA was synthesized using a cDNA Synthesis Kit (Invitrogen). qRT-PCR was performed with the SYBR Green Master Mix (Tiangen, Beijing, China). The primers used were as follows: HIF-1*α*, forward primer 5′-TCACCACAGGACAGTACAGGATGC-3′ and reverse primer 5′-CCAGCAAAGTTAAAGCATCAGGTTCC-3′; VEGFA, forward primer 5′-AGGAGGGCAGAATCATCACG-3′ and reverse primer 5′-CAAGGCCCACAGGGATTTTCT-3′; and GAPDH, forward primer 5′-TTACTCCTTGGAGGCCATGTGGGC-3′ and reverse primer 5′-ACTGCCACCCAGAAGACTGTGGATGG-3′.

### 2.5. Nucleoprotein Extraction and Western Blot Analysis

Subcellular fractionation was performed as per the manufacturer's instructions (Thermo Scientific, San Jose, CA, USA). Protein concentrations of cellular or nuclear extracts were determined using a bicinchoninic acid (BCA) assay kit (Bio-Rad Laboratories, Inc.). In brief, equal amounts (20-25 *μ*g) of protein fractions of lysate were resolved using SDS-polyacrylamide gel electrophoresis (SDS-PAGE), transferred to PVDF membranes (Millipore, USA), and immunoblotted as previously described in our study [23].

### 2.6. Gene Transfection and RNAi

The method was described in our previous study [5]. Silencing of pVHL shRNA was acquired by way of lentiviral transduction of the following specific shRNA vectors, purchased from Santa Cruz Biotechnology: pVHL shRNA (sc-36816-V) and scramble shRNA control (sc-108080).

### 2.7. Statistical Analyses

The data were analyzed with SPSS software (version 21.0, Chicago, IL, USA) and expressed as the mean ± standard deviation (SD). Chi-squared, Kaplan-Meier, and Pearson's correlation analyses were used to analyze TMA data. Student's *t*-tests were used for comparisons between 2 groups, and one-way analysis of variance was used for comparisons between multiple groups. *P* < 0.05 was considered to indicate statistically significant results.

## 3. Results

### 3.1. Expression and Correlation between COX-2 and HIF-1*α* in HCC Specimens

**Figure 1:**
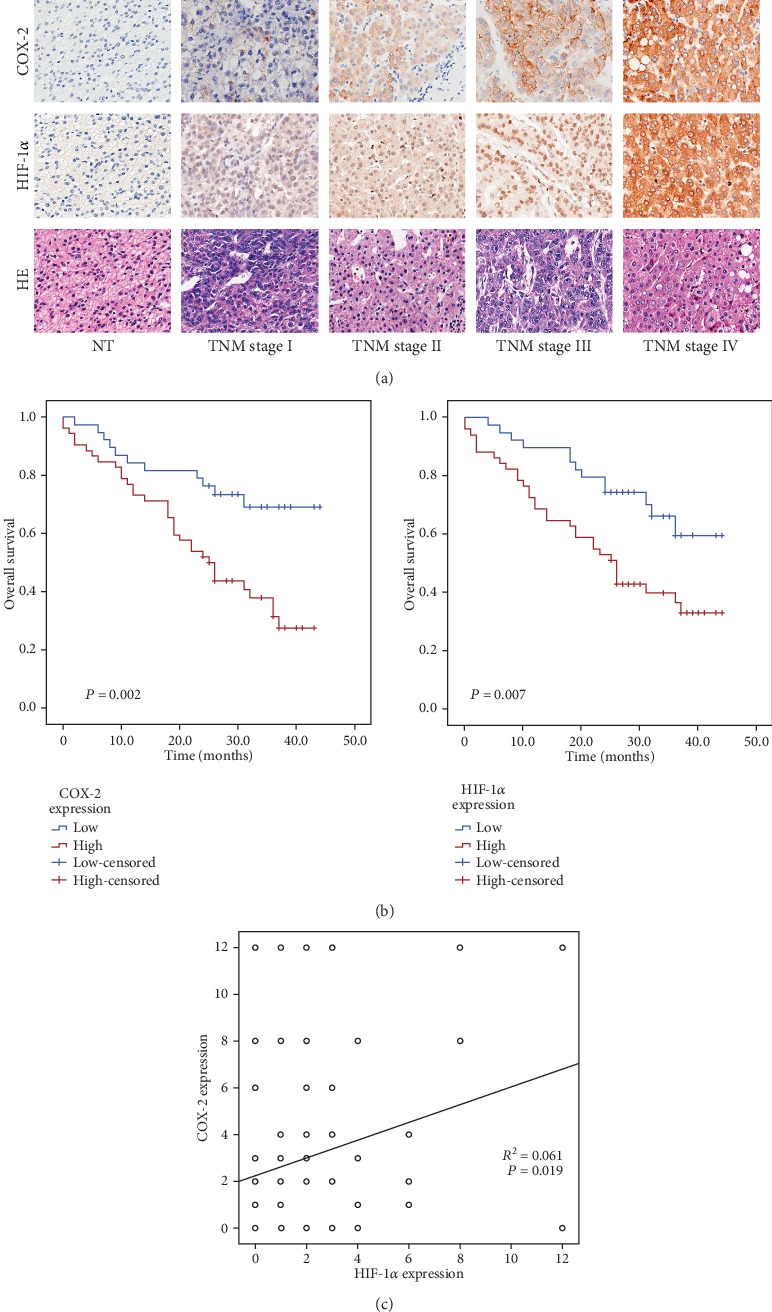
COX-2 and HIF-1*α* expressions in HCC tissues. (a) IHC staining of COX-2 and HIF-1*α* and HE in HCC tissue (magnification ×200). (b) High COX-2 or HIF-1*α* expressions correlate with poorer overall survival (*P* = 0.002 and *P* = 0.007, respectively, log-rank test). (c) Positive correlation between COX-2 expression and HIF-1*α* level (Pearson's correlation, *R*^2^ = 0.061, *P* = 0.019).

We first examined expression of COX-2 and HIF-1*α* in HCC tissues in a TMA. As shown in Figure 1(a), the IHC staining of COX-2 and HIF-1*α*, with hematoxylin and eosin (HE), were classified as TNMI-IV. The clinicopathological distribution features of COX-2 are presented in Table 1. There was no significant correlation between COX-2 expression and clinicopathological variables including patient age, sex, histology grade, and lymph node metastasis (Table 1). The association between patient survival and COX-2 or HIF-1*α* expression was measured through the Kaplan-Meier analysis and log-rank test, respectively. The data demonstrated that compared with low expression of COX-2 or HIF-1*α*, the overall survival in HCC patients with high expression of COX-2 or HIF-1*α* has a downward trend (*P* = 0.002 and *P* = 0.007, respectively, Figure 1(b)).

The univariate Cox proportional hazards regression model was applied in order to evaluate the crude hazard ratios (HRs) of COX-2 expression or each clinicopathological variable on patient survival. According to the univariate Cox regression analyses, COX-2 expression was closely related to overall survival (*P* = 0.003, Table 2). Multivariate analysis was conducted, and the significant factors are summarized in Table 2 so as to further confirm the prognostic value of COX-2. Expression of COX-2 was an independent prognostic marker according to the Cox regression model. Moreover, a positive correlation between COX-2 expression and the level of HIF-1*α* was found in accordance with the results, regardless of nuclear or cytoplasmic localization (Pearson's correlation, *R*^2^ = 0.061, *P* = 0.019, Figure 1(c)).

### 3.2. Meloxicam Exerts an Antitumor Effect under Hypoxic Conditions in 2 HCC Cell Lines

**Figure 2:**
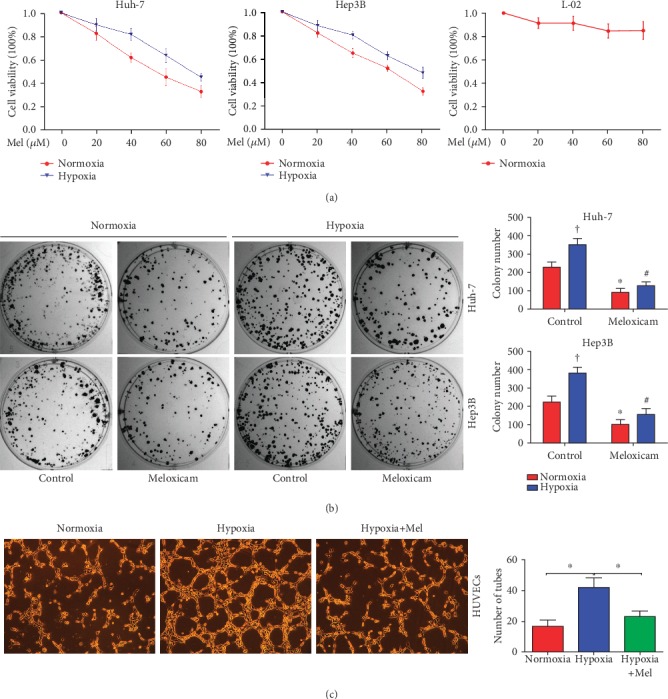
Meloxicam has potent antitumor efficacy in 2 HCC cell lines but no toxicity in normal human liver cell lines *in vitro*. (a) Two HCC cell lines and the normal liver cell line, L-02, were incubated with meloxicam at various concentrations (0 to 80 *μ*M) for 24 h in normoxic or hypoxic conditions, and then, the cell viability was determined by CCK-8 assay. (b) Representative colonies formed are shown in the left panel and the quantified results in the right panel. ^†^*P* < 0.05 and ^∗^*P* < 0.05, compared with cells untreated with meloxicam under normoxic conditions; ^#^*P* < 0.05, compared with cells untreated with meloxicam under hypoxic conditions. (c) HUVECs were incubated for the tube formation assay under normoxic or hypoxic conditions with or without meloxicam (80 *μ*M) (magnification × 100). ^∗^*P* < 0.05. The experiments were performed in triplicate. Data are presented as mean ± SD.

Meloxicam, a selective COX-2 inhibitor, exerts extensive antitumor effect on various malignant tumors [24, 25]. Therefore, in our study, it was hypothesized that meloxicam could inhibit HCC cell proliferation and angiogenesis, especially under hypoxic conditions. Huh-7 and Hep3B cells were exposed to meloxicam for 24 h under normoxic or hypoxic conditions, and cell proliferation was determined utilizing the CCK-8 and colony formation assay. As shown in Figure 2(a), meloxicam significantly inhibited cell viability under normoxic conditions and weakened the hypoxia-induced proliferation capability in both HCC cells. However, meloxicam only mildly affected the normal human liver cell line: L-02. The results of the colony formation assay were consistent with the cell viability assay. A notable increase induced by hypoxia in the clonogenic survival of HCC cells could be reversed by meloxicam (Figure 2(b)). Next, we investigated the effect of meloxicam on angiogenesis. Human umbilical vein endothelial cells (HUVECs) were incubated with or without meloxicam (80 *μ*M) for 24 h under normoxic or hypoxic conditions. It was found that capillary-like tube formation was remarkably inhibited by meloxicam under hypoxic conditions (Figure 2(c)).

### 3.3. Meloxicam Overcomes Hypoxia-Induced Apoptotic Resistance Requiring Caspase Activities in HCC Cells

**Figure 3:**
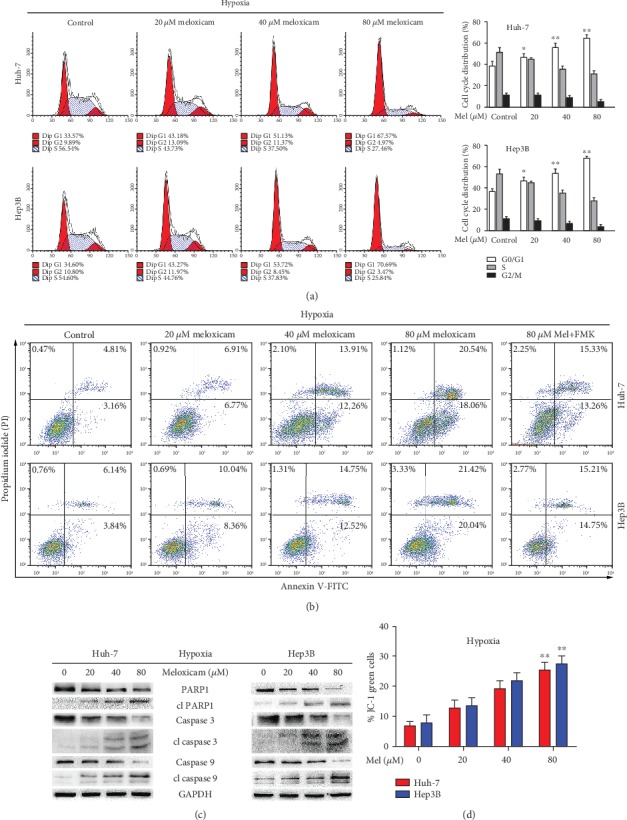
Meloxicam induces caspase-dependent apoptosis in human HCC cells under hypoxic conditions. (a, b) Cells were treated with meloxicam (0 to 80 *μ*M) or meloxicam (80 *μ*M)+Z-VAD-FMK (50 *μ*M) under hypoxic conditions for 24 h. The cell cycle and apoptosis were analyzed by FACS flow cytometry. ^∗^*P* < 0.05 and ^∗∗^*P* < 0.01 vs. control. (c) Protein levels of PARP1, caspase-3, and caspase-9 were detected by Western blot analysis. Levels of GAPDH served as a loading control. (d) Disruption of mitochondrial membrane potential (*ΔΨm*) was determined by JC-1 analysis. ^∗∗^*P* < 0.01 vs. control. Data are presented as means ± SD of three independent experiments.

An analysis of the cell cycle and apoptosis of Huh-7 and Hep3B cells under hypoxic conditions was performed by flow cytometry (FACS). As shown in Figures 3(a) and 3(b), meloxicam markedly arrested both Huh-7 and Hep3B cells in the G1 phase and increased cellular apoptosis in a concentration-dependent manner. Caspase activation is considered one of the apoptosis mechanisms in COX-2 inhibitor-treated tumor cells [26]. In the present study, we used Western blot to detect expression of PARP1, caspase-3, and caspase-9 after Huh-7 and Hep3B cells were exposed to meloxicam under hypoxic conditions. As shown in Figure 3(c), meloxicam dose dependently decreased the level of full-length PARP1, procaspase-3, and procaspase-9 and strengthened the cleavage of PARP1, caspase-3, and caspase-9 in HCC cells. In addition, Z-VAD-FMK, a pancaspase inhibitor, notably decreased meloxicam-induced apoptosis (Figure 3(b)). During apoptosis, another important intracellular event is the occurrence of the disruption of mitochondrial membrane potential (*ΔΨm*). We found, by JC-1 analysis, that meloxicam notably weakened *ΔΨm* in both HCC cells, which suggested that meloxicam causes disruption of *ΔΨm*, thereby triggering caspase-dependent apoptosis (Figure 3(d)).

### 3.4. Meloxicam Downregulates HIF-1*α* Transcription Activity and Expression in a VHL-Dependent Manner

**Figure 4:**
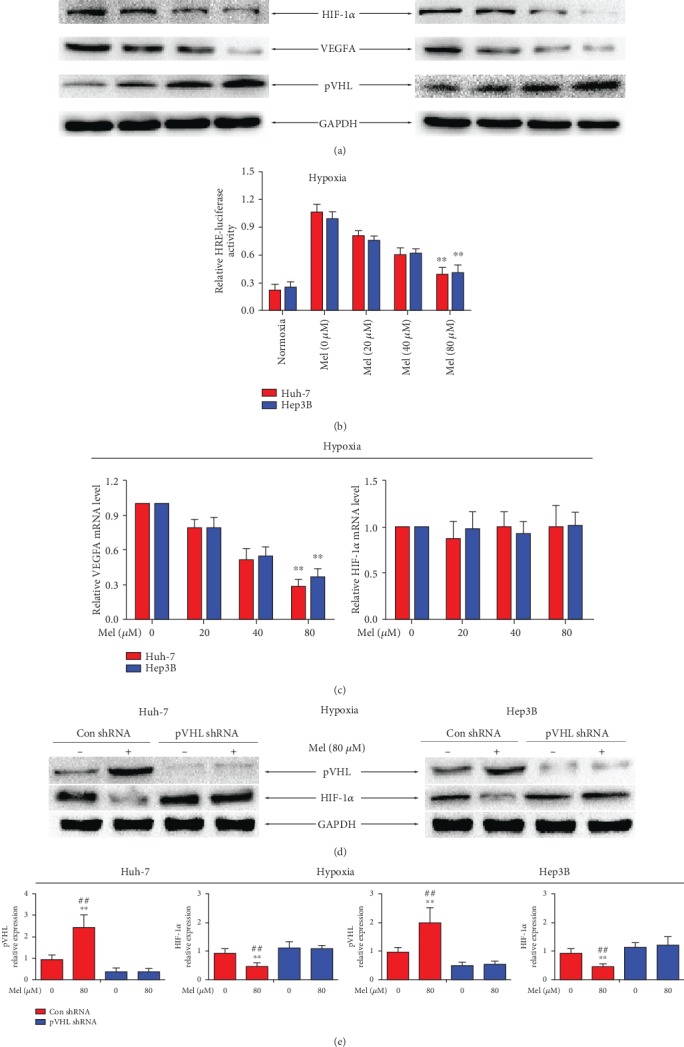
Meloxicam downregulates HIF-1*α* transcriptional activity and expression in a pVHL-dependent manner. (a) Cells were treated with meloxicam (0 to 80 *μ*M) for 24 h under hypoxic conditions. Protein levels of HIF-1*α*, VEGFA, and pVHL were detected by Western blot analysis. Levels of GAPDH served as a loading control. (b) An HRE-dependent reporter assay was used to detect the effect of meloxicam on HIF-1*α* transcriptional activity. ^∗∗^*P* < 0.01 compared with untreated controls in hypoxia. (c) Total RNA was extracted, and HIF-1*α* and VEGFA mRNA expressions were analyzed by qRT-PCR. ^∗∗^*P* < 0.01 vs. control. (d, e) pVHL-silenced or control cells were treated with or without meloxicam (80 *μ*M) for 24 h under hypoxic conditions. Protein expressions of pVHL and HIF-1*α* were determined by Western blot. Levels of GAPDH served as a loading control. ^∗∗^*P* < 0.01 compared with the meloxicam-untreated control shRNA group; ^##^*P* < 0.01 compared with the meloxicam- (80 *μ*M) treated pVHL shRNA group. Data are representative of 3 independent experiments.

Accumulated evidence has demonstrated that HIF-1*α* has a dominant role in tumor progression, and HIF-1*α*-regulation of expression of VEGFA is regarded as the main inducer of angiogenesis [27–29]. In the current study, we hypothesized if meloxicam could inhibit HIF-1*α* expression and its target gene VEGFA. To answer this question, the two HCC cell lines were treated with meloxicam (0 to 80 *μ*M) for 24 h under hypoxic conditions. It was found that meloxicam significantly decreased the level of HIF-1*α* and VEGFA protein expression compared to the control group (Figure 4(a)). To investigate whether meloxicam could inhibit the transcriptional activity of HIF-1*α* in Huh-7 and Hep3B cells, a hypoxia-responsive reporter including a luciferase gene with a hypoxia response element (HRE) was introduced. As shown in Figure 4(b), the HIF-1*α* transcriptional activities of Huh-7 and Hep3B cells exposed to meloxicam were both weakened under hypoxic conditions. qRT-PCR results indicated that the extent of VEGFA mRNA was also suppressed when treated with meloxicam. However, interestingly, it was found that incubation with meloxicam did not attenuate the extent of HIF-1*α* mRNA, which suggested that meloxicam may regulate HIF-1*α* at posttranscriptional levels but not at the transcriptional level (Figure 4(c)).

As a negative regulator of HIF-1*α*, pVHL has an important role in cellular oxygen sensing through ubiquitination and subsequent proteasomal degradation [7]. The loss of pVHL results in the accumulation and translocation of HIF-*α* into the nucleus, which subsequently activates the transcription of HIF target genes to participate in important oncogenic pathways, such as angiogenesis [30, 31]. Here, we explored whether meloxicam was involved in the process of HIF-1*α* degradation through pVHL upregulation. As shown in Figure 4(a), meloxicam notably upregulated the expression of pVHL in a concentration-dependent manner. To further ascertain the effect of pVHL in meloxicam-regulated suppression of HIF-1*α*, a lentivirus-mediated pVHL shRNA to knock down pVHL was introduced in Huh-7 and Hep3B cell lines which were defined as Huh-7-pVHL shRNA cell and Hep3B-pVHL shRNA cell, respectively. Western blot results showed that pVHL silencing could attenuate the suppression of HIF-1*α* by meloxicam, which suggested that meloxicam downregulates HIF-1*α* signaling by augmenting pVHL expression under hypoxic conditions (Figures 4(d) and 4(e)).

### 3.5. Meloxicam Attenuates HIF-1*α* Nuclear Translocation and Promotes the Proteasomal Degradation of HIF-1*α*

**Figure 5:**
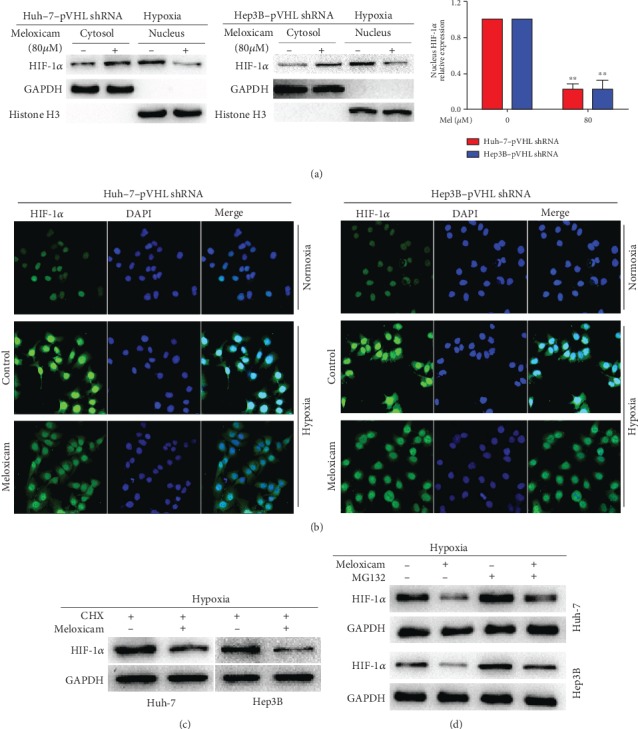
Meloxicam reduces HIF-1*α* nuclear translocation and promotes the proteasomal degradation of HIF-1*α*. (a) Cytoplasmic and nuclear fractions were extracted, and expression levels of HIF-1*α* were analyzed by Western blot. Levels of GAPDH and histone H3 served as a loading control. ^∗∗^*P* < 0.01 vs. control. (b) Meloxicam sequesters HIF-1*α* in the cytoplasm and reduces HIF-1*α* nuclear translocation under hypoxic conditions as detected by IF staining (magnification ×200). (c) Cells were exposed to CHX (10 *μ*g/ml) in the presence or absence of meloxicam (80 *μ*M) under hypoxic conditions for 24 h. Protein levels of HIF-1*α* were detected by Western blot analysis. (d) Cells were exposed to meloxicam (80 *μ*M) with or without MG-132 (10 *μ*M) under hypoxic conditions for 24 h. Protein levels of HIF-1*α* were detected by Western blot analysis. Levels of GAPDH served as a loading control. Data are representative of 3 independent experiments.

The effect of meloxicam in significantly decreasing HIF-1*α* transcriptional activity urged us to further investigate whether meloxicam could affect HIF-1*α*'s subcellular localization for hypoxia-induced nuclear translocation protecting HIF-1*α* against downregulation by way of pVHL as previously reported [32]. Western blotting results revealed that meloxicam repressed the nuclear localization of HIF-1*α* compared with empty vector controls in both Huh-7-pVHL shRNA and Hep3B-pVHL shRNA cell lines (Figure 5(a)). In order to further confirm the effect of meloxicam on reducing HIF-1*α*'s nuclear accumulation and sequestering it in the cytoplasm, immunofluorescent (IF) staining was utilized and the results showed that meloxicam treatment altered the nuclear localization of HIF-1*α* in Huh-7-pVHL shRNA and Hep3B-pVHL shRNA cell lines whose cytosolic localization was increased and nuclear accumulation was suppressed under hypoxic conditions (Figure 5(b)). Based on these findings, we are eager to further explore the mechanisms through which meloxicam downregulates HIF-1*α*. Proteasomal degradation plays a crucial role in cellular protein turnover [33, 34]. Cycloheximide (CHX) is an antifungal antibiotic that inhibits protein synthesis in eukaryotes. Here, we used CHX to inhibit de novo protein synthesis in Huh-7 and Hep3B cell lines. In this way, changes in the expression of HIF-1*α* will mainly reflect its protein degradation. We found that the intensity of HIF-1*α* in the presence of CHX was dramatically diminished by meloxicam treatment which implied that meloxicam may be involved in the degradation of HIF-1*α* (Figure 5(c)). Next, we utilized a specific proteasome inhibitor, MG132, to further test the hypothesis that meloxicam targets HIF-1*α* for proteasomal degradation. It was found that MG132 treatment in the presence of meloxicam suppressed downregulation of HIF-1*α* which revealed that meloxicam could induce HIF-1*α* proteasomal degradation in a hypoxic environment (Figure 5(d)).

### 3.6. Meloxicam Arrests Tumor Growth and Angiogenesis In Vivo

**Figure 6:**
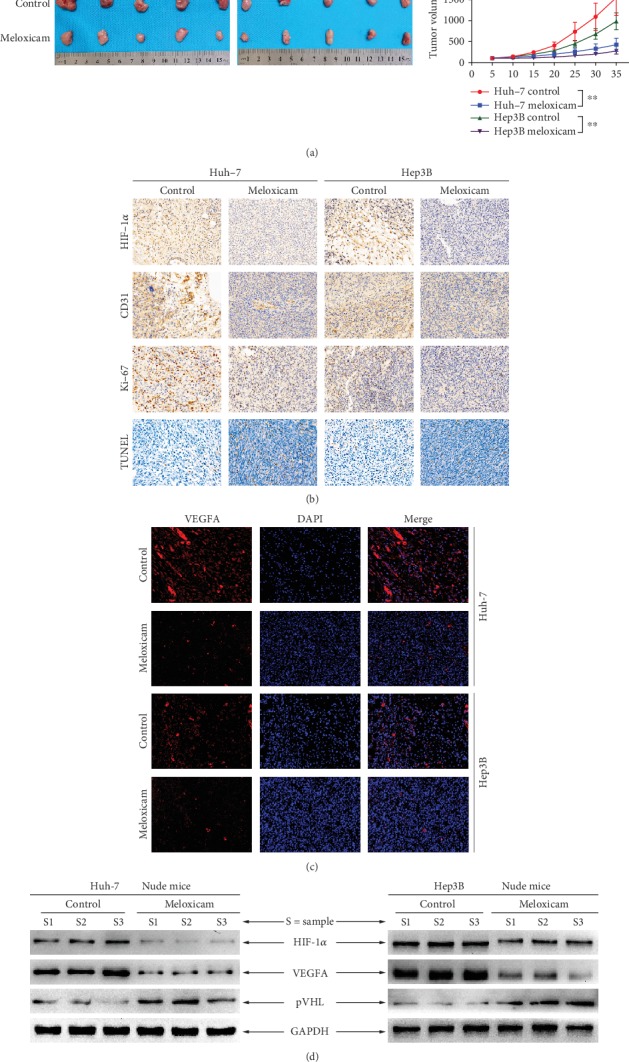
Meloxicam arrests tumor growth *in vivo*. (a–d) Nude mice bearing established Huh-7 and Hep3B tumors were treated with 0.9% NS or meloxicam (30 mg/kg) for 35 . (a) Tumors from mice after tumor implantation are shown. The average tumor volume for each group was calculated. ^∗∗^*P* < 0.01. (b) Representative images of tumor sections stained with antibodies against HIF-1*α*, Ki-67, CD31, and the TUNEL agent (magnification × 200). (c) Expression and distribution of VEGFA in xenograft tumor tissues were determined by immunofluorescent photomicrography (magnification, ×200). (d) Western blot analysis of Huh-7 and Hep3B cell-derived tumors treated with 0.9% NS or meloxicam (30 mg/kg) for expressions of HIF-1*α*, VEGFA, and pVHL. GAPDH was measured as the loading control.

Considering meloxicam's superior antitumor effects *in vitro*, we investigated whether meloxicam could inhibit tumor development *in vivo*. As shown in Figure 6(a), meloxicam notably suppressed growth of Huh-7 and Hep3B xenograft tumors. Next, HIF-1*α* as well as hallmarks of growth and angiogenesis was detected by IHC and IF analysis, which revealed downregulation in HIF-1*α*, Ki-67, and CD31 in tumor tissues treated with meloxicam, whereas TUNEL staining showed opposite results (Figure 6(b)). The result of IF analysis revealed the suppression of expression of VEGFA in meloxicam-treated groups (Figure 6(c)). The data of Western blot showed that meloxicam treatment remarkably blocked the level of HIF-1*α* and VEGFA, while enhancing pVHL expression in Huh-7 and Hep3B cell-derived tumors (Figure 6(d)).

## 4. Discussion

Given that hypoxic HCC cells are proangiogenic and antiapoptotic and that the HIF-1*α* signaling pathway has a crucial role in regulating cellular adaptation to hypoxia, our study investigated responses of Huh-7 and Hep3B cell lines to meloxicam, a COX-2-selective inhibitor, which has been considered a potential candidate for targeting the HIF-1*α*-VEGF axis [15], and investigated the potential mechanism involved in regulating these responses. The present study initially utilized TMA technology and IHC to explore expression of COX-2 and HIF-1*α* in HCC. The data indicated a positive correlation between COX-2 expression and the HIF-1*α* level and high COX-2 or HIF-1*α* expression in connection with poor prognosis in HCC patients. In the *in vitro* experiment, it was demonstrated that meloxicam not only suppressed cell growth and angiogenesis but also caused disturbance of mitochondrial membrane potential (*ΔΨm*), leading to caspase-dependent apoptosis under hypoxia. However, there was no significant cytotoxicity in normal human liver cell lines treated with meloxicam. We also found that meloxicam had the ability of inhibiting tumor growth in subcutaneous HCC mouse models *in vivo*.

In previous studies, it was found that HIF-1*α* was notably stabilized in malignant tumor cells under hypoxic conditions, along with upregulating its target gene, VEGFA, which is considered the principal inducer of angiogenesis [35]. This current study showed that both the protein levels of HIF-1*α* and VEGFA were decreased when treated with meloxicam. But qRT-PCR analysis showed that the extent of HIF-1*α* was only mildly changed after being exposed to meloxicam. To investigate whether meloxicam could affect the transcriptional activity of HIF-1*α*, a reporter plasmid was introduced and the results revealed that meloxicam treatment results in reduced HIF-1*α* transcription activity. These data implied that meloxicam decreased HIF-1*α*'s transcription activity but not via its transcriptional level and therefore, meloxicam may be a posttranscriptional regulator of HIF-1*α*. Moreover, the level of HIF-1*α* in the presence of CHX, an inhibitor of protein synthesis, was significantly reduced by meloxicam treatment. MG132, which is a proteasomal inhibitor, exposed to meloxicam prevented the degradation of HIF-1*α*. These data revealed that meloxicam treatment alters the nuclear localization of HIF-1*α* and promotes its degradation. Therefore, we thought an important possibility that might explain the mechanisms of inhibition of tumor growth is that HIF-1*α* cytoplasmic-nuclear trafficking and proteasomal degradation by meloxicam could counteract hypoxia-regulated drug resistance.

**Figure 7:**
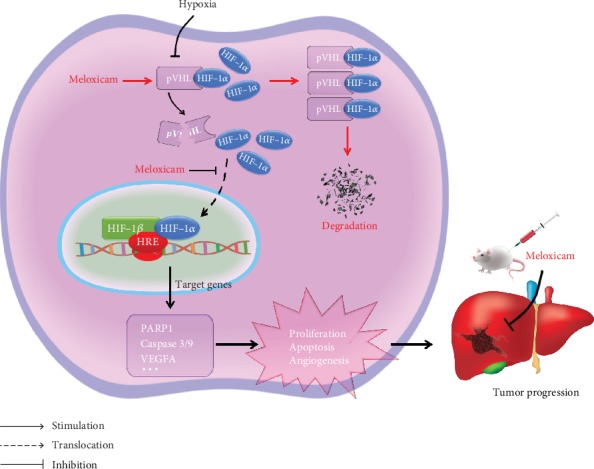
Schematic model depicting the possible mechanisms of apoptotic resistance and angiogenesis induced by hypoxia and the underlying molecular mechanisms through which meloxicam overcomes the HIF-1*α* signals in HCC.

Previous studies reported that the pVHL is one of the negative regulators of the HIF transcription factor [36–38]. Thus, modulating the extent of pVHL expression under hypoxic conditions may offer an effective therapy for HCC. The current work indicated that meloxicam inhibits the HIF-1*α* signaling pathway and reduces its target gene VEGFA through upregulating the pVHL protein both *in vitro* and *in vivo* whereas VHL silencing suppresses meloxicam-mediated HIF-1*α* downregulation. In conclusion, our results provide direct and strong evidence that the HIF-1*α* signaling pathway and its target genes can function as a powerful force for HCC cells to remain alive in a hypoxic environment. We further demonstrated that blocking HIF-1*α* by meloxicam could overcome angiogenesis and apoptosis resistance in HCC (Figure 7). These data provide strong evidence that the COX-2-specific inhibitor exhibits promising potential for the treatment of HCC, but further clinical investigation is still required.

## Figures and Tables

**Table 1:** Correlation between COX-2 expression and clinicopathological characteristics of HCC patients.

	Variables	COX-2 expression		Total	*χ* ^2^	*P* value
		Low	High			
Age (year)					0.729	0.393
	≤54	17	28	45		
	>54	21	24	45		

Sex					0.178	0.673
	Female	6	10	16		
	Male	32	42	74		

Grade					1.966	0.161
	I/II	30	34	64		
	III	8	18	26		

T stage					5.024	0.025^∗^
	T1/T2	27	25	52		
	T3/T4	9	24	33		
	Dull					

N stage						1
	N0	36	47	83		
	N1	0	0	0		
	Dull					

M stage						1
	M0	36	47	83		
	M1	0	1	1		
	Dull					

TNM stage					5.024	0.025^∗^
	Ι/II	27	25	52		
	III/IV	9	24	33		
	Dull					

Tumor size					4.819	0.028^∗^
	≤5 cm	22	18	40		
	>5 cm	16	34	50		

^∗^Statistically significant (*P* < 0.05).

**Table 2:** Univariate and multivariate analyses of the factors correlated with overall survival of HCC patients.

Variables	Univariate analysis			Multivariate analysis		
	HR	95% CI	*P* value	HR	95% CI	*P* value
Expression	2.780	1.406-5.494	0.003^∗^	2.373	1.101-5.112	0.027^∗^
Sex	2.253	0.885-5.737	0.089			
Grade	2.034	1.158-3.572	0.013^∗^	1.414	0.741-2.696	0.293
Age	0.985	0.954-1.018	0.366			
T stage	2.687	1.593-4.530	≤0.001^∗^	3.338	0.726-15.338	0.121
N stage			1			
M stage			1			
TNM stage	2.616	1.488-4.600	0.001^∗^	0.449	0.079-2.559	0.367
Tumor size	1.085	1.036-1.137	0.001^∗^	1.044	0.967-1.128	0.269

^∗^Statistically significant (*P* < 0.05).

## Data Availability

The data used to support the findings of this study are available from the corresponding author upon request.
